# Neurons contribute to pathology in a mouse model of Krabbe disease in a cell-autonomous manner

**DOI:** 10.1371/journal.pbio.3001706

**Published:** 2022-07-06

**Authors:** Pedro Brites, Monica M. Sousa

**Affiliations:** 1 Neurolipid Biology Group, Instituto de Biologia Molecular e Celular (IBMC), Instituto de Investigação e Inovação em Saúde (i3S), University of Porto, Porto, Portugal; 2 Nerve Regeneration Group, Instituto de Biologia Molecular e Celular (IBMC), Instituto de Investigação e Inovação em Saúde (i3S), University of Porto, Porto, Portugal

## Abstract

This Primer explores the implications of a PLOS Biology showing that in vivo, neurons (not only myelinating glia) are primary effectors of disease progression in Krabbe disease; the neuron-specific animal model described allows an unprecedented opportunity to investigate the neuronal-autonomous component of this disorder.

## Teaser

In this issue of *PLOS Biology*, Kreher and colleagues show in a mouse model that in vivo, neurons and not only myelinating glia are primary effectors of disease progression in Krabbe disease. The neuron-specific model generated allows the unprecedented capacity to investigate the neuronal autonomous component of this disorder.

In Krabbe disease, also known as globoid cell leukodystrophy, mutations in β-galactosylceramidase (GALC), a lysosomal enzyme responsible for the catabolism of myelin galactolipids, lead to the accumulation of its substrates galactocerebroside and psychosine [1]. Whereas galactocerebroside builds up inside macrophages and microglia giving rise to globoid cells, psychosine accumulates in myelinating glia culminating in demyelination and is therefore considered the main culprit of the disorder. The estimated incidence of Krabbe disease ranges from 1:250,000 in the United States of America to 1:100,000 in Europe and 1:100 in some communities with high consanguinity. The most common form of the disorder is infantile, although late onset and adult forms of Krabbe disease also occur. In infants, the disorder is generally diagnosed before 6 months of age, and symptoms are very severe including irritability, stiffness, loss of vision and hearing, seizures, and paralysis. In the absence of treatment, disease progression is very rapid with death occurring within 2 to 3 years given organ failure as a result of demyelination. In late onset patients, symptoms have a more variable onset and disease progression is usually slower. The Twitcher mouse, a naturally occurring model of Krabbe disease (with a W355* nonsense mutation that abolishes enzymatic activity), mimics the infantile form of the disorder. In Twitcher mice, in agreement with the ubiquitous nature of *Galc* expression, psychosine accumulates in myelinating glia and neurons [2]. As a possible consequence of psychosine accumulation in neurons, axonal swellings [2] and axonal loss [3] occur in the Twitcher nervous system before the onset of demyelination, supporting a myelin-independent dying-back neuropathy. The existence of neuronal deposits of α-synuclein in Twitcher mice and human Krabbe samples [4], and the fact that induced neurons from Krabbe patient’s fibroblasts present axonal defects [5], further contributed to support that neurons are an important neglected cell type primarily affected in this disorder. However, in the above models, given the ubiquity of Galc, the final proof of the neuron-autonomous nature of the defects observed remains to be provided, as one cannot exclude the contribution of alternative cells to the neuronal pathology, nor a putative prior conditioning of cultured neurons.

**Fig 1:**
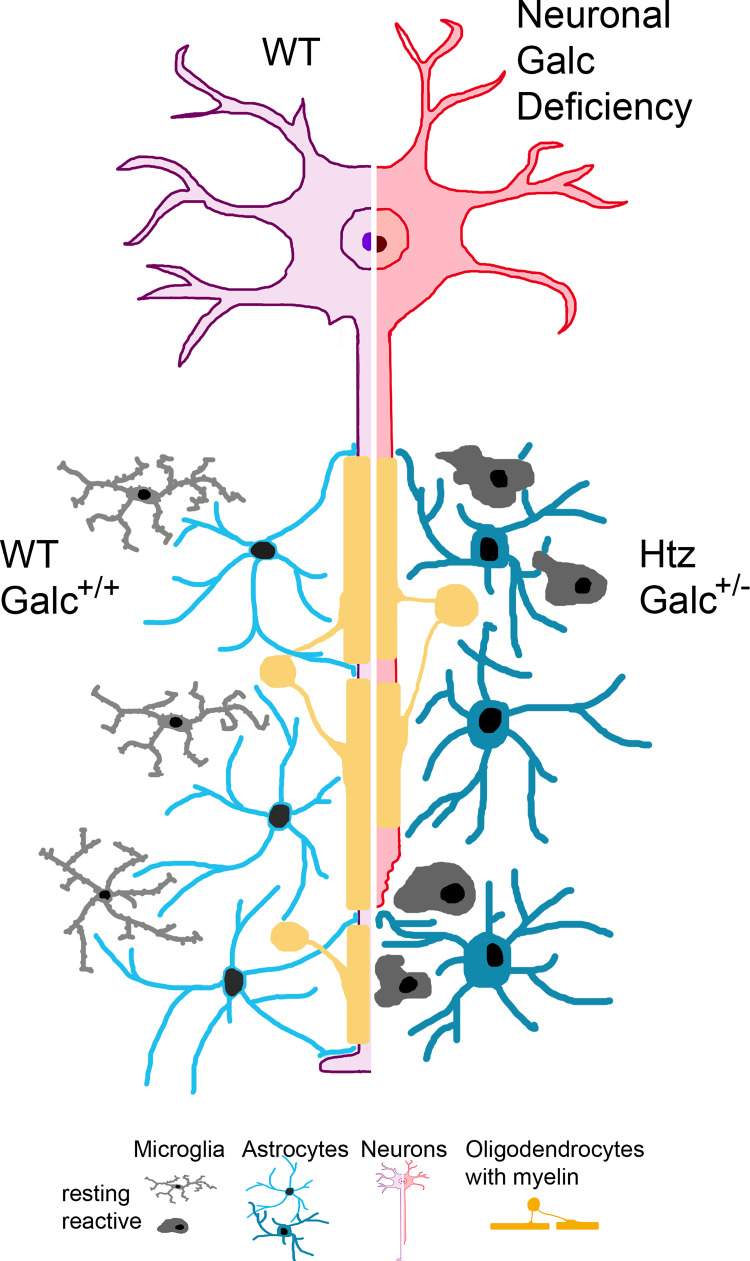
Affected cells in the CNS of Syn1Cre:Galc^f/−^ mice. Representation (left) of a WT neuron within a CNS populated with myelinating oligodendrocytes, resting microglia, and astrocytes. Representation (right) of a Syn1Cre:Galc^f/−^ neuron within the CNS populated by Galc^+/−^ heterozygous (Htz) cells. The neuronal Galc deficiency causes distal degeneration of axons and a generalized astrocytic and microglial response. Activated microglia (appear more amoeboid-shaped) and activated astrocytes (hypertrophic) are observed in the CNS of Syn1Cre:Galc^f/−^ mice, possibly due to the ongoing neurodegeneration. The neuronal Galc deficiency also affects Galc^+/−^ oligodendrocytes, as they display reduced amounts of MBP and MAG and thinner myelin sheaths. CNS, central nervous system; MAG, myelin-associated glycoprotein; MBP, myelin basic protein; WT, wild-type.

In this issue of *PLOS Biology*, Kreher and colleagues use the recently generated conditional *Galc* floxed mouse [6] to eliminate Galc from neurons and investigate the existence of a neuron-autonomous role for Galc. In the genetic background of *Galc* heterozygosity (*Galc*^f/−^), neuronal ablation was achieved by Synapsin 1–mediated expression of cre recombinase. The homozygous loss of Galc in neurons (Syn1Cre:*Galc*^f/−^) is shown to be sufficient to induce a neurological phenotype with growth and motor-coordination defects. The analysis of Syn1Cre:*Galc*^f/−^ mice revealed that psychosine accumulates in the nervous system where neuronal and axon degeneration, neuroinflammation (astrocytosis and microgliosis), and a mild reduction in myelination occur (Fig 1). Thus, this publication underscores that neuronal Galc is essential for normal central nervous system (CNS) homeostasis and directly contributes to the pathogenesis of Krabbe disease. A limitation of this study is however the use of a generalized *Galc* heterozygosity background (*Galc*^+/−^), in which all murine cells only have one functional *Galc* allele. Although *Galc*^+/−^ mice do not develop an overt phenotype or pathology, and express normal levels of several myelin components, *Galc* heterozygosity causes a reduction in Galc activity [6], impaired microglial function, and defective repair of myelin damage [7]. Thus, studies using *Galc*^f/f^ mice in a wild-type (WT) background would serve to fully decouple effects of loss of Galc activity in neurons from possible mild consequences of *Galc* heterozygosity.

The findings of Kreher and colleagues underscore the need to further understand the specific cellular and molecular mechanisms of GALC function in neurons. A substantial body of data already shows the cell-specific deleterious effects of psychosine in this cell type. Psychosine can intercalate in membrane lipid rafts disrupting their structure and, as a consequence, impairing lipid-raft mediated signaling, including critical pathways for neuronal development and function such as PKC, Erk, and AKT-GSK3β [3,8,9]. In addition, psychosine accumulation in neurons also impairs endocytosis and axonal transport [3,8], whose tightly regulated balance is critical for normal neuronal biology and function. It is also essential to consider that neuronal-derived psychosine may influence neighboring nonneuronal cells by being carried in extracellular vesicles, as recently shown in Twitcher mice [10]. Beyond psychosine, galactosylceramide (that is also GALC substrate) and its biosynthetic enzyme (UDP galactosyltransferase 8A - UGT8a), are generally viewed as oligodendrocyte specific. In this respect, it is possible that neuronal galactosylceramide might be transferred to neurons from neighboring myelinating cells. Finally, one should take into account that if axonal survival is critically dependent on supporting glia, healthy functional axons are also required for the maintenance of fit myelinating glia. By showing that the neuronal-specific deletion of GALC in neurons leads to neuroinflammation and mild myelin loss, Kreher and colleagues’ findings reinforce the need to further focus on the neuron-glial crosstalk in the context of leukodystrophies.

In summary, in this issue of *PLOS Biology*, the study of Kreher *and colleagues* opens new windows of action in Krabbe disease by highlighting that in vivo neurons are an important primary cellular target in this disorder. Although originally classified as a myelin disorder—leukodystrophy—in which the involvement of CNS and peripheral nervous system (PNS) myelin mediated a secondary neurodegeneration, there is now mounting evidence of an additional neuron-autonomous effect behind Galc deficiency. It is possible that similar cell-autonomous mechanisms unrelated to myelinating glia operate in other leukodystrophies, especially for those in which the disease-causing gene shows generalized expression in multiple cell types. Thus, not only new therapeutic strategies focused on targeting and correcting primary neuronal defects but also further knowledge on cell-specific primary effects caused by the accumulation of toxic substrates in different cell types of the nervous system are needed in Krabbe disease and also in other leukodystrophies.

## Abbreviations

CNS: central nervous system
GALC: galactosylceramidase
PNS: peripheral nervous system
WT: wild-type
